# Study on the influence of shot peening strengthening before shot peen forming on 2024-T351 aluminum alloy fatigue crack growth rate

**DOI:** 10.1038/s41598-023-32616-2

**Published:** 2023-03-31

**Authors:** Guowei Li, Zhicheng Dong, Tianhao Luo, Heyuan Huang

**Affiliations:** 1 School of Civil Aviation, Northwestern Polytechnical University, Xi’an, China; 2 School of Aeronautics, Northwestern Polytechnical University, No. 127, Youyi West Road, Beilin District, Xi’an, Shaanxi China; 3 Institute of Optics and Electronics, Chinese Academy of Sciences, Chengdu, China; 4 Research & Development Institute of Northwestern Polytechnical University in Shenzhen, Shenzhen, Guangdong China

**Keywords:** Aerospace engineering, Mechanical engineering

## Abstract

It is sparse and inconclusive that research on the subject whether the fatigue life of the structure will be reduced by shot peening strengthening before shot peen forming (S + F), and this study investigates accordingly. First, the crack growth rate test of the machine-processing plate and shot peening strengthening before shot peen forming plate demonstrate that both plates’ final crack growth rate and length are similar. However, the test shows the “fluctuation phenomenon” of crack growth rate and the “intersection phenomenon” in the Paris curve. This study is based on a self-developed simulation plugin for crack growth paths. The results verify that “fluctuation” causes the differential distribution of the overall stress intensity factor in the strengthened (4.5% increase compared to machine-processing) and formed (9.8% decrease compared to machine-processing) crater areas of the shot peening strengthening before shot peen forming plate. Comparing to the full coverage strengthening area, the forming area (only 30% coverage) in the early stage of growth as well as the gain amplitude of the residual stress in the late stage of growth gradually decrease and tend to be the same as that of the machine-processing, as validated by the “intersection phenomenon”.

## Introduction

2024-T351 aluminum alloy, with its lightweight and high strength characteristics, has been widely used in the main bearing components of aerospace aircraft^1–5^. Under the action of long-term alternating loads, these structural parts will produce minor cracks and further expand, eventually leading to structural failure. Therefore, aeronautical components are usually post-treated by the shot peening strengthening process, which effectively slows down the crack growth rate by introducing residual stresses on the material’s surface and causing plastic deformation^6^^7^, thereby improving the fatigue life of the structure^8,9^. At present, researchers have carried out a large number of studies on shot peening strengthening process parameters such as shot peening coverage^10–12^, speed^13,14^ and pressure^15^, specimen surface roughness^16^^17^, geometric surface parameters^18^, etc., forming a relatively complete shot peening strengthening process system.

In recent years, the integrated forming technology of aircraft large wall plates has become more mature. Shot peening forming, possessing the advantages of flexibility, low cost, and no need for dedicated moulds, has been widely used in forming and preparing of aircraft wings and fuselage overall wall panels^19^. The shot peening forming process drives the elastic extension of the inner layer of the material through the plastic deformation of the surface material in the shot peening forming process, and makes use of the difference in deformation between the two to achieve the effect of integral molding in two directions^20^. Similar to the shot peening strengthening process, the researchers conducted a large number of studies on the shot peening forming process parameters such as projectile size, shot peening forming time and pressure^21,22^ , and specimen thickness^19,23^, prestress and edge conditions^24^. Wang, Zhang, et al.^25,26^. also used the finite element method to predict the fatigue life of the specimen accurately. In addition, numerous studies have proved that the current widespread usage of low coverage large-size projectiles for its capability to improve the forming efficiency and increase the surface roughness of the specimen, thereby reducing the fatigue performance of the model^27^.

To solve the structural fatigue performance degradation caused by the forming process, the researchers studied the influence of the process of first shot peening and the ultrasonic shot peening process on the fatigue performance of the specimen. For example, Takahiro et al. found that the forming and strengthening shot peening process is applied on the same side, in which the curvature of the specimen gradually increases with the application of shot peening strengthening, while the opposite side is adverse^28^. However, after strengthening in different positions, the residual stresses near the surface of the specimen increase. Wang et al.^29^ studied the effect of projectile size on the fatigue performance of the sample after the F + S process. They found that the 1.2 mm projectile increased the DFR (detail fatigue rating) benchmark value of the piece by 31%. Wang et al.^30^ studied the effects of different plate thicknesses and shot peening forming coverage on the curvature of the specimen. The curvature increased with the escalation of plate thickness and decreased with the addition of coverage, but gradually reduced the degree of reduction. A Rajesh et al.^31^ used RSM (response surface method) experimental design and found that the fatigue strength of the specimen was increased by up to about 34% at a 5.699 MPa shot peening pressure and a distance of 30 mm. Moreover, Men et al.^32,33^, who studied the effect of ultrasonic amplitude on shot peening intensity, saturation time and roughness, concluded that the intensity value and amplitude were negatively correlated with the amplitude, while the saturation time and amplitude were positively correlated with the amplitude. The cut-off length of 122 μm could better control the roughness. Zhang et al.^34^ studied the influence of ultrasonic parameters on bending deformation and surface properties. The results showed that improving the speed and diameter of the firing pin, increasing the number of shot blasting, and reducing the shot peening distance were conducive to improving the chordal deformation as well as obtaining greater surface hardness and residual pressure stress. While these new shot-peening processes have increased the fatigue performance in numerical simulations and small specimen testing, the introduction of other processes after forming negatively impact the forming effect.

The above studies show that the F + S process can improve the fatigue strength of the specimen. Still, it will change the curvature of the model, which adversely affects the structural forming accuracy^35–37^, making the process more complicated and less controllable^38^. Therefore, to investigate the effect of the S + F process on the fatigue performance of the structure, the crack growth rate of the 2024 aluminum alloy prepared by mechanical processing (MP) and the S + F process were compared by experiments in this paper. By using the extended finite element method (XFEM) which avoids the re-gridding of the mesh, we have significantly saved the running time and computational cost and improved the computational accuracy have. Furthermore, coupling the XFEM with damage extrapolation and virtual crack closure (VCCT), a finite element model of the crack growth rate of the specimen was established. After that, the effect of the S + F process on the crack growth rate of 2024 aluminum alloy could be clarified by comparing and analyzing the test and finite element simulation results. Finally, based on the numerical method, a crack extension length calculation tool was developed to reveal the “fluctuation phenomenon” formation mechanism in the test curve and the “intersection phenomenon” in the fitted curve of the crack growth rate test results.

## Experiment

### Basic mechanical properties test

**Figure 1:**
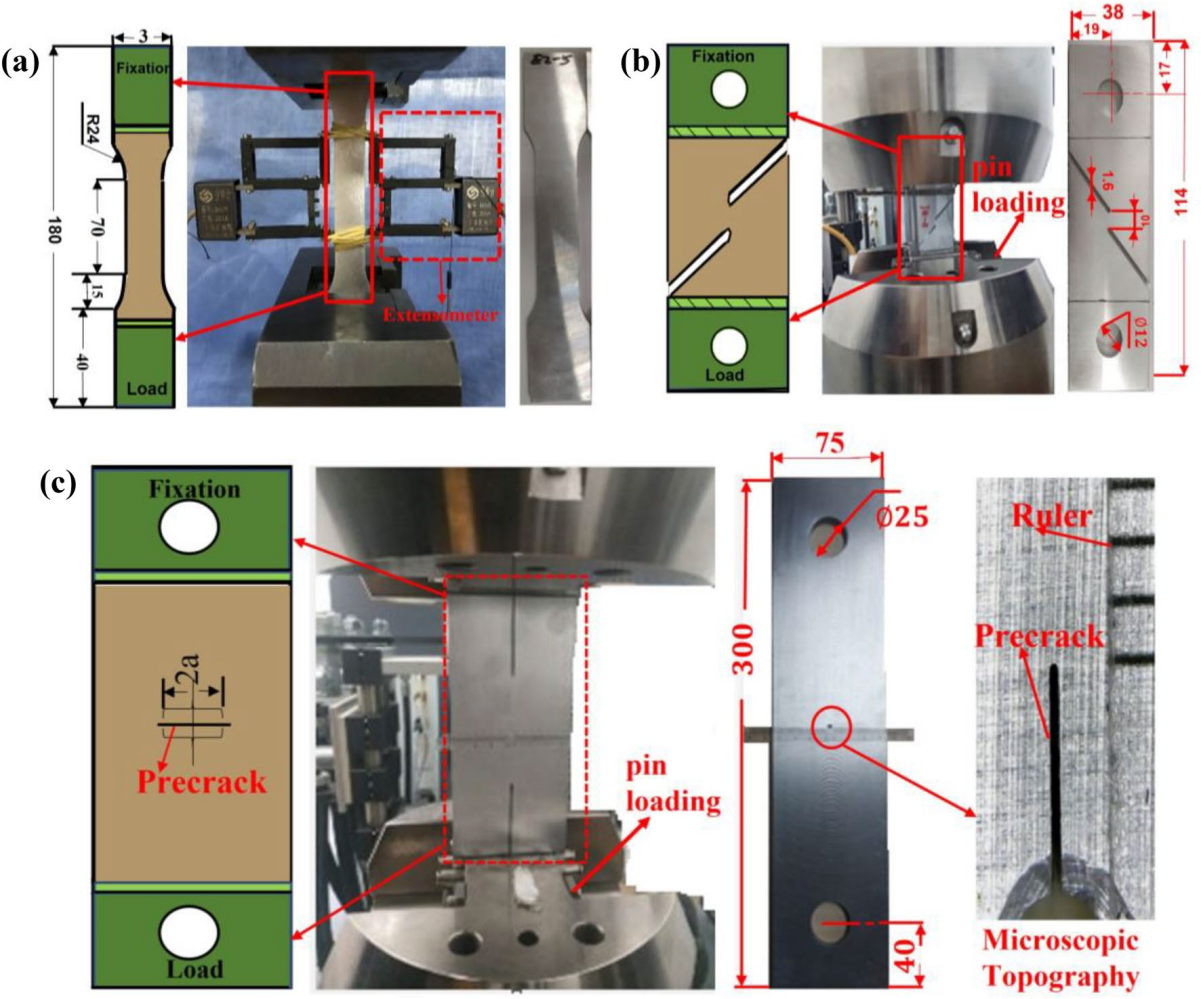
Schematic diagram of the test. (**a**) tensile test (**b**) shear test (**c**) Crack growth rate test. (unit:mm).

Tensile performance tests and shear performance tests for MF plate and S + F process plate in accordance with ASTM E8 M-08^42^ and ASTM F1160^43^ test standards are conducted. The test material is 2024-T351 aluminum alloy, the specimens are standard test pieces, the tensile models are 5 mm thick, and the shear specimens are 3.8 mm wide. The tests schematic are shown in Fig. 1a,b. In the tests, both ends of the specimens were fixed by the chuck of the testing machine. The lower chuck was moved downward at a rate of 2 mm/min under the control of the testing machine to achieve tensile and shear loading. A static strain analysis system was used to acquire of strain, with six samples of each type.

### Residual stress test

This section introduces residual stress measurement tests according to ASTM E915^44^ test standards for large crater areas of shot peening forming and only shot peening strengthening enhancement areas. The test was conducted through a μ-360 s X-ray diffractometer whose tube operating voltage and current were 30 kV and 3 mA. The target material used was chromium target, the measurement method was cosα method, the sample distance was 65 mm, and the measurement range was about 2 mm in diameter spot. The test position is at the center of the sample, and the residual stress is tested at the surface, 50 μm, 100 μm, 200 μm, 400 μm, 600 μm, and 800 μm positions along the length of the sample, respectively. Using the method described above for direct measurement of residual stresses at different locations in the surface layer. To carry out the residual stress test in the depth direction, brine electrolytic etching was used for stripping corrosion, with a water ratio of 1:4, and the residual stress was measured after layer-by-layer electrolytic corrosion.

### Crack growth rate test

To investigate the influence of the S + F process on the crack growth rate of 2024 aluminum alloy, a crack growth rate test was carried out according to ASTM E647^45^ test standard to obtain the fatigue life (N) and crack growth length (a) of the specimens, providing data support for the subsequent calculation and analysis of crack growth rate (da/dN) and overall stress intensity factor (∆k). The test was divided into two groups (MP and S + F) of six pieces each and the test schematic diagram is shown in Fig. 1c, with six samples of each class. The dynamic fatigue testing machine is used for loading. The chuck of the testing machine fixes both ends of the specimen where the lower chuck applies a constant amplitude alternating load F = 16 kN, the stress ratio is 0.06, the waveform is set to a sine wave, and the test frequency is 5 Hz.

The incremental polynomial method performs a local fit derivative to determine the fitted values of fatigue crack growth rate and crack length^39^. For any test data point i and 3 points before and after each, a total of 7 continuous data points, the following quadratic polynomials are used for fitting and differentiation.

1 $$\widehat{{a}_{i}}={\mathrm{b}}_{0}+{\mathrm{b}}_{1}\left(\frac{{\mathrm{N}}_{\mathrm{i}}-{\mathrm{d}}_{1}}{{\mathrm{d}}_{2}}\right)+{\mathrm{b}}_{2}{(\frac{{\mathrm{N}}_{\mathrm{i}}-{\mathrm{d}}_{1}}{{\mathrm{d}}_{2}})}^{2}$$

2 $${a}_{i-n}\le \alpha \le {a}_{i+n}$$

$$-1\le \left(\frac{{N}_{i}-{d}_{1}}{{d}_{2}}\right)\le 1$$

$${d}_{1}=0.5({N}_{i+n}{+N}_{i-n})$$

$${d}_{2}=0.5({N}_{i+n}{-N}_{i-n})$$

The fitted value $\widehat{{a}_{i}}$ is the fitted crack length at ${N}_{i}$ corresponding to the number of cycles, the coefficients ${b}_{0}, {b}_{1}, {b}_{2}$ are the regression parameters determined by the least-squares method in the interval of Eq. (2), the parameters ${d}_{1}$ and ${d}_{2}$ are the input data through the transformation, and the crack growth rate at ${N}_{i}$ is derived from Eq. (1) as follows:

3 $$\frac{\widehat{da}}{dN}=\frac{{b}_{1}}{{d}_{2}}+\frac{2{b}_{2}({N}_{i}-{d}_{1})}{{{d}_{2}}^{2}}$$

The value of ∆k corresponding to the da/dN value is calculated from the fitted crack length ${a}_{i}$ corresponding to ${N}_{i}$.

4 $$\Delta K=\frac{\Delta P}{B}\sqrt{\frac{\pi \alpha }{2W}\mathit{sec}\frac{\pi \alpha }{2}}$$

where $\Delta P={P}_{max}-{P}_{min}$, $\mathrm{\alpha }=2a/W$, $a={a}_{0}+{a}_{i}$ among which $a$ is the calculated crack length and ${a}_{0}$ is the sum of the specimen notch length and opening radius (2 mm), P is the applied load, B is the plate thickness (6 mm), W is the plate width (75 mm).

In the steady-state growth phase of fatigue cracks, the rate of crack growth is usually described using the Paris power function equation.

5 $$\frac{da}{dN}=C\Delta {K}^{n}$$

## Numerical methods

This section focuses on the fatigue crack growth simulation of 2024 aluminum alloy plate after pre-cracking. The numerical methods for simulating crack growth mainly include finite difference method, meshless method, boundary element method, and finite element method (FEM), among which FEM is widely used in numerical simulations due to its numerous advantages in dealing with crack tip stress field. However, FEM, when dealing with strong discontinuities such as crack extension, requires re-gridding at each extension step, leading to increased workload and error accumulation. Therefore, in this paper, we use XFEM to avoid the refinement of the crack tip mesh during the simulation. The method characterizes the crack discontinuity by introducing enrichment terms to the displacement functions of the nodes on the unit being penetrated and the unit containing the crack tip. The displacement functions of XFEM are as follows:

6 $$\sum_{i=1}^{m}{N}_{i}(x)\left[{u}_{i}+H(x){a}_{i}+{\sum }_{a=1}^{4}{F}_{a}(x){b}_{i}^{a}\right]$$

where ${N}_{i}$ is the shape function, ${u}_{i}$ is the degree of freedom, ${a}_{i}$ is the extra degree of freedom to model the jump across crack faces, $H(x)$ is the jump function, ${F}_{a}(x)$ is the enrich function, and ${b}_{i}^{a}$ is the addeddegree of freedom associated with ${F}_{a}(x)$. The jump function can be written as the following:

7 $$\left\{\begin{aligned}H\left(x\right) = 1, if\left(x-{x}^{*}\right)*n\ge 0\\ H\left(x\right)=-1, otherwise\end{aligned}\right.$$

where $x$ is an arbitrary point near the crack face, ${x}^{*}$ is the closest point to $x$ located on the crack face, and $n$ is the normal vector of cracks at ${x}^{*}$. The crack tip enrichment function is taken as follows:

8 $${F}_{\alpha }\left(x\right)=\left[\sqrt{r}\mathrm{sin}(\frac{\theta }{2}),\sqrt{r}scos(\frac{\theta }{2}),\sqrt{r}sin\theta \mathrm{sin}(\frac{\theta }{2}),\sqrt{r}\mathrm{sin}(\frac{\theta }{2})\mathrm{cos}(\frac{\theta }{2})\right]$$

where r and θ are the local polar coordinates at the crack tip. After the displacement model is constructed, the basic control equations for the extended finite element solution can be derived according to the principle of virtual work, just like the conventional FEM, after which the overall stiffness matrix, the load matrix, and then the nodal displacements are solved. XFEM allows the crack to appear at any location in the cell. The crack-free elements are solved by the same Gaussian integration method as in FEM, while subdividing elements penetrated by cracks and containing the crack tips. These elements are then divided into several small cells and the boundaries of the sub-cells are made consistent with the crack geometry interface, changing the integral over the whole cell into the sum of the integrals over all sub-cells.

Considering the effect of residual stress field, ABAQUS firstly implements the calculation of steady-state cycling of the structure through the Direct cyclic analysis cyclic step, combining the material damage extension criterion and Fourier technique to obtain the cyclic solution. During each cyclic loading process, direct cyclic analysis is used for immediate structural response cyclic analysis. The method is based on constructing a displacement function u(t) to describe the structural response of the structure at all time points (t) throughout the cyclic loading cycle (T), thus enabling the performance of the Low-cycle Fatigue Analysis of the large structure.

### Finite element model

#### Model parameters

First, we establish the geometric model of the crack growth feature area and consider the model’s symmetry. Then, the right side of the feature area is taken for analysis under the symmetry boundary condition for constraint on the central symmetry plane. At the beginning of the process, the initial crack size is 2 mm. One end of the flat plate is fixed, and the other end is applied with constant amplitude alternating load where F is 16 kN, stress ratio is 0.06, the waveform is set a to sine wave. XFEM is used and the mesh size is selected as 1 mm. In order to obtain more accurate and high precision crack growth data, the cells on the crack growth path are refined. The meshing results are shown in Fig. S1.

In the crack growth rate test, there is a significant change in the rate when the crack tip expanded to the vicinity of the forming crater. Therefore, considering the effect of the geometry of the crater on the crack growth, for the crater unit, a very low stiffness C3D8R unit is used to replace the area vacated by the crater. At the same time, the damage criterion based on the energy release rate was changed to the Max Damage maximum nominal strain criterion to ensure crack growth in the crater region.

#### Introduction of residual stress

**Figure 2:**
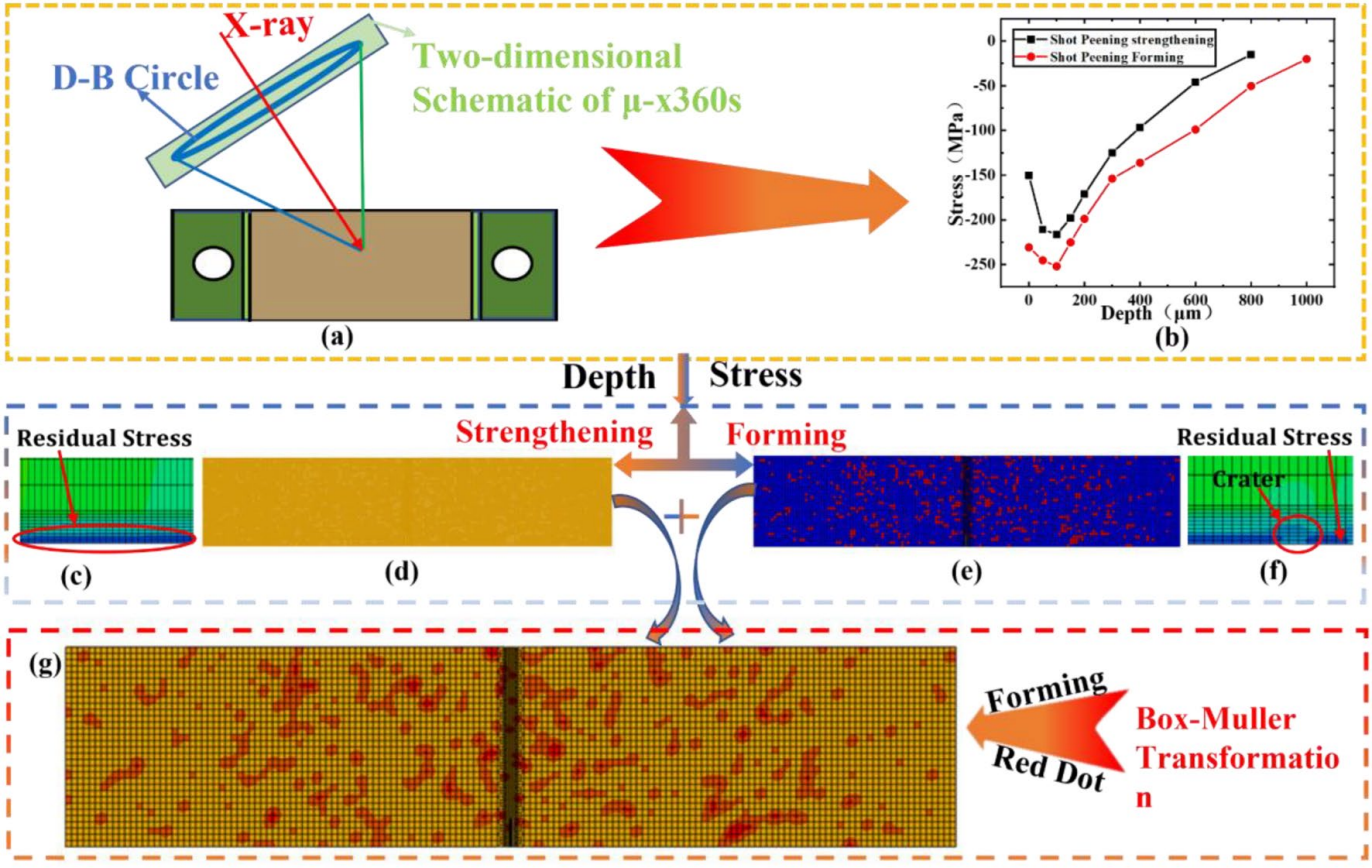
Residual stress distribution after S + F: (**a**) μ-360sX; (**b**) Residual stress test results; (**c**) depth direction stress; (**d**) surface layer stress; (**e**) surface layer stress; (**f**) depth direction stress; (**g**) residual stress introduction results.

Using the SIGINI subroutine, the residual stresses in each element are set by coordinates. Then, the residual stress field is polynomially fitted in Matlab and the residual stress layer on the model surface is defined by a function definition. In order to obtain a more accurate residual stress distribution, the surface elements need to be refined along the thickness direction. The thickness of each layer is set to 0.1 in the range of 0–0.5, the thickness of surface layer is set to 5.5–6, and the thickness of each layer element is set to 0.5 in the range of 0.5–5.5. The shot peening strengthening process is fully covered, so only the thickness of the stress distribution is programmed. For the formed crater area, the position coordinates of the formed crater are generated by the Box-Muller transformation, which creates random variables that follow the uniform distribution and implements the position coordinates in FORTRAN. A residual stress field is established on the surface of the crack propagation specimen and is balanced by static load steps. The residual stress is introduced as shown in Fig. 2 below.

### Finite element method

#### Low-cycle fatigue analysis methods

Low-period fatigue analysis is performed using the direct cycle method and combined with the extrapolation of injury technique. The crack tip energy release rate (G) is calculated by the VCCT and the Power criterion in ABAQUS. The crack growth rate is calculated based on G. Since the software only utilizes $\Delta G$ to investigate the fatigue crack growth, the relationship between $\Delta G$ and $\Delta K$ can be written as follows for plane strain conditions:

9 $$\Delta G = \frac{{1 - v^{2} }}{E}(\Delta K_{{\text{I}}}^{2} + \Delta K_{{{\text{II}}}}^{2} ) + \frac{1 + v}{E}\Delta K_{{{\text{III}}}}^{2}$$

where $\Delta {K}_{\mathrm{ I }}^{2}$, $\Delta {K}_{\mathrm{ II }}^{2}\,\mathrm{ and}$ ${\Delta K}_{\mathrm{ III }}^{2}$ are mode I, II and III $\mathrm{stress\,intensity\,factor\,ranges}$, respectively. The fatigue crack growth rate is described quantitatively through the Paris formula^40^.

**Figure 3:**
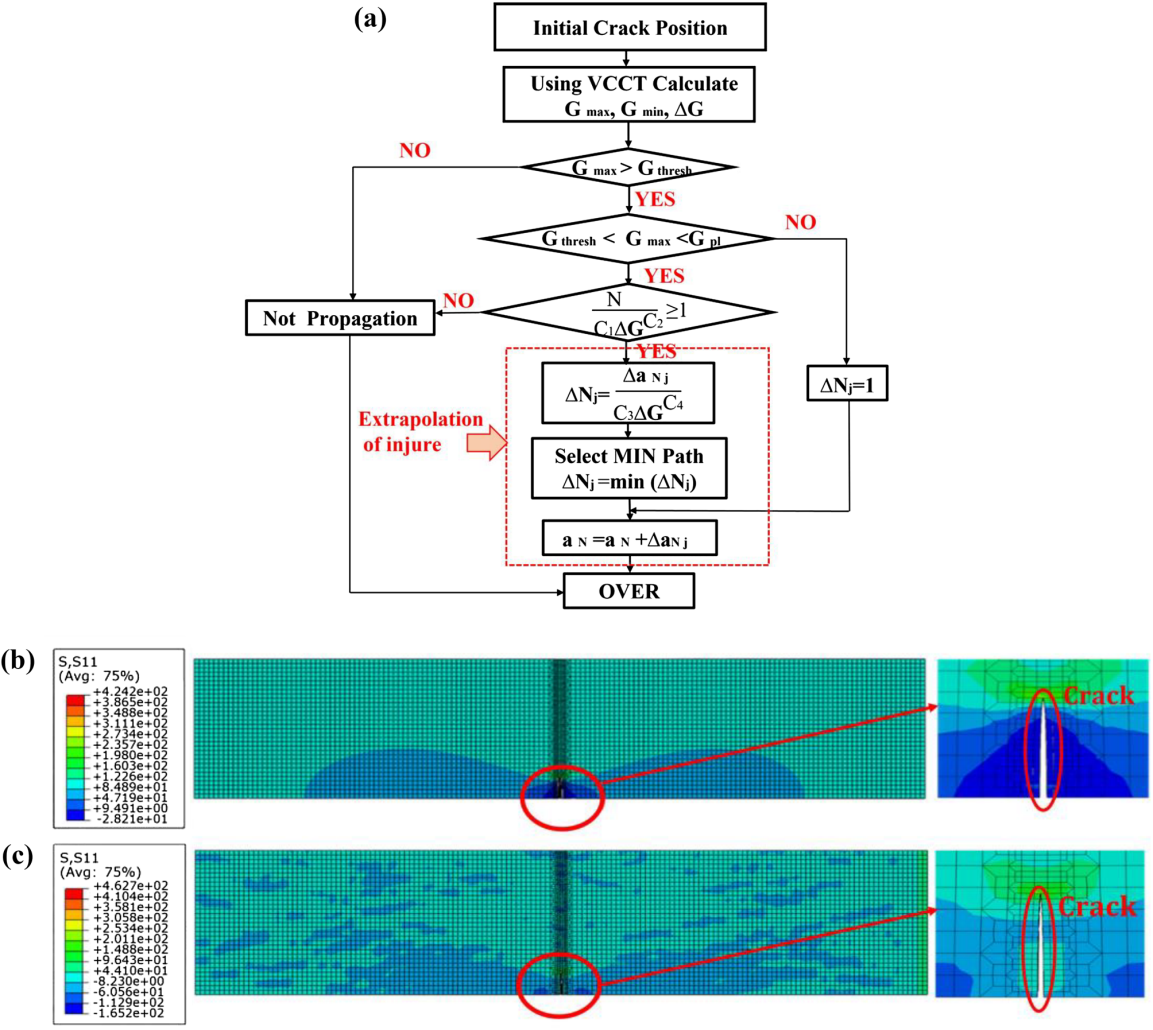
Crack growth finite element process and results: (**a**) Analysis process (**b**) MP Results; (**c**) S + F Results.

The fatigue growth analysis process is shown in Fig. 3a below.

Where C_1_ and C_2_ are material parameters of the fatigue crack initiation, C_3_ and C_4_ are material parameters based on the energy release rate, ${G}_{max}$ and ${G}_{min}$ are the energy release rates corresponding to the peak and valley values of the load, ${G}_{\mathrm{thresh}}$ and ${G}_{\mathrm{pl}}$ are the upper and lower nodes of the classical Paris region, $\Delta {N}_{j}$ is the cycle period required to extend to the leading edge of the crack, and $\Delta {a}_{Nj}$ is the spacing between the crack tip points before and after the growth.

#### Crack length calculation plug-ins

In ABAQUS, the crack state can be described by PHILSM (Initial Crack Orientation) and PSILSM (Specify Crack Plane Position)^46,47^. The crack length output is not directly provided, so this paper develops a plug-in for the variation of crack length with cycle. In data post-processing, the path of the crack within the cell is located by the PSILSM variable value. Meanwhile, in ABAQUS extended finite elements, the crack tip cell cannot stay in the inner cell, but only on the boundary. The crack is approximated as a straight line in the cell, so only the positions of the two endpoints of the crack in the cell on the boundary need to be obtained to get the crack length in the cell. In addition, the whole crack length can be obtained by the cumulative summation of multiple cells.

Through the finite element result file, all PHILSM valuable nodes are read and the keyframes are filtered by the dichotomous method until they approach the crack extension frame. Then, all the crack extension keyframes in the whole cycle are extracted. The crack length is accumulated to get the final crack length. Specifying the initial crack coordinate position and the total number of output frames of the result file, the crack length corresponding to each cycle can be automatically calculated, of which results are saved in matrix form. After that, the variation curve with the number of cycles is generated. Figure S2 shows the plug-in and finite element results.

## Results

### Test results

The basic mechanical property parameters of MP and S + F can be obtained from the basic mechanical property test. And it provides the parameter input for the later numerical simulation. The test results are shown in Table 1.

**Table 1:** Basic mechanical property parameters test results.

	E (MPa)	${\sigma }_{p0.2}$(MPa)	${\sigma }_{t1.5}$(MPa)	μ	S
Average value (MP)	69,300	313.50	362.05	0.33	306.34
Discrete coefficient (MP)	1.70%	0.80%	0.20%	1.92%	9.20%
Average value (S + F)	69,120	323.81	361.12	0.32	288.85
Discrete coefficient (S + F)	0.70%	9.00%	4.40%	3.04%	2.04%

PS: E is tensile modulus, ${\sigma }_{p0.2}$ is yield strength, ${\sigma }_{t1.5}$ is tensile strength, μ is Poisson's ratio, S is shear strength.

Through the residual stress testing test, we found that the residual stress depth and size of the secondary impact area of the formed projectile were increased to varying degrees. Moreover, the depth of the residual stress layer was increased by about 24.5%, and the maximum residual stress level was increased by 16.1%. The test results are shown in Fig. 2b. The shot peening process will cause uneven plastic deformation of the specimen, thus forming a certain thickness of the reinforced layer, which may cause a high residual stress to form within the reinforced layer. The thickness and size of the residual stress mainly depends on the size of the projectile we used. The larger the diameter of the projectile, the greater the depth and value of the plastic strain and residual stress layer in the specimen. Meanwhile, the shot peening strengthening process mainly uses small sized projectiles and the shot peening forming process mainly uses large sized projectiles, which results in deeper and higher levels of residual stresses in the shot peening forming process compared to the strengthening process, leading to the difference in residual stress test results as shown in the Fig. 2b.

**Figure 4:**
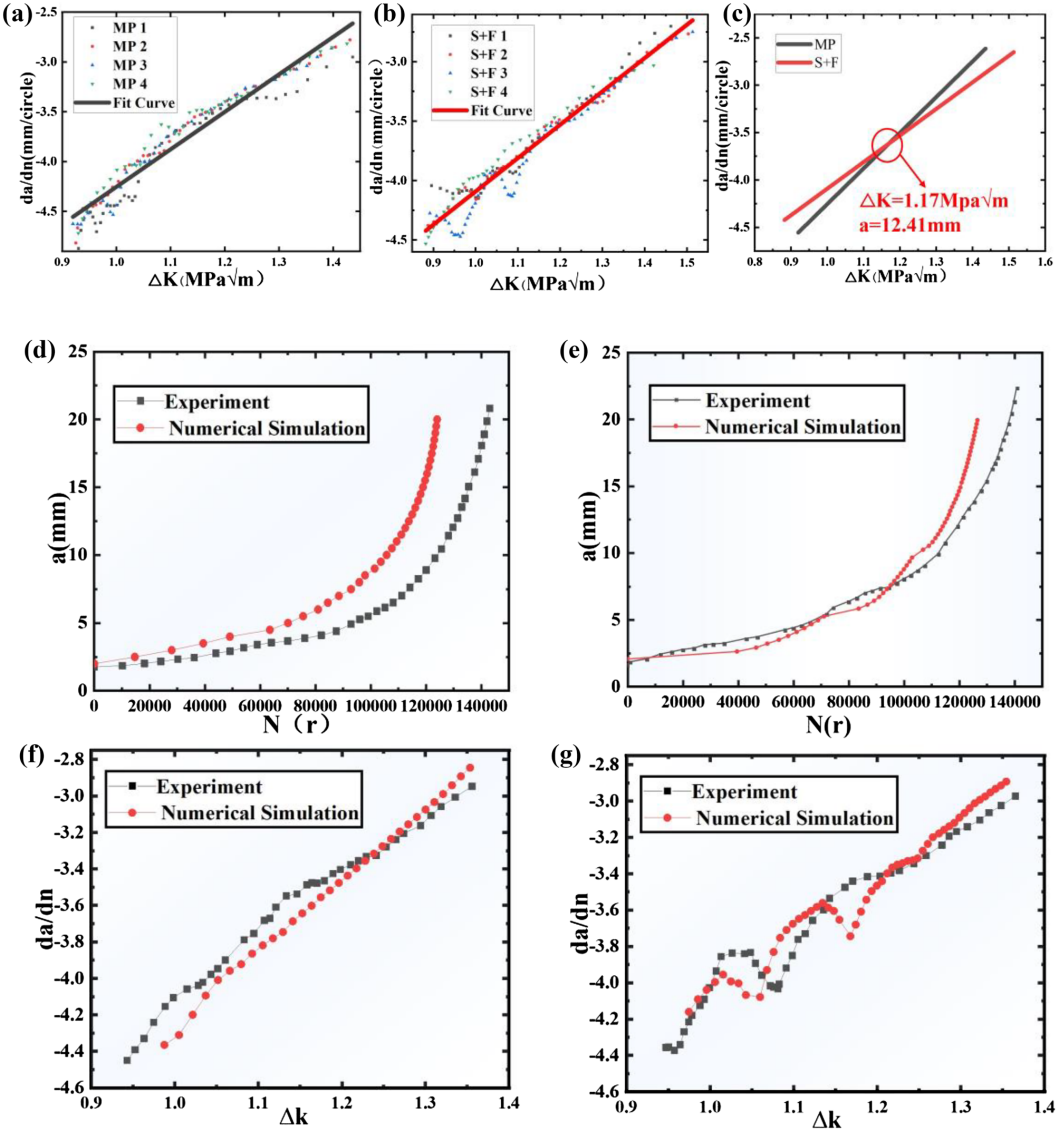
Experiment and numerical simulation results: (**a**) MP fitting results; (**b**) S + F fitting results; (**c**) “intersection phenomena”; (**d**) MP validation results; (**e**) S + F validation results; (**f**) MP Paris results; (**g**) S + F Paris results.

The crack growth rate test results are logarithmically solved by Eq. (5) and fitted to the results. We find that the intersection of the two lines $\mathrm{is} \Delta \mathrm{K}=1.17\mathrm{Mpa}\sqrt{m}$ and a = 12.41 mm. Up to this point, the growth rate of the S + F plate is greater than the MP plate. However, beyond this point, the crack growth rate of the S + F plate drops below the MP plate. The test results are shown in Fig. 4a,b,c.

### Numeric method validation

The finite element calculation result file was first post-processed to extract the variation of crack growth length with fatigue cycle through the crack length calculation plug-in and then compared with the test results. The results are shown in Fig. 4d,e. Data processing is performed on the extracted crack length and fatigue period. The crack growth rate is calculated form the energy release rate of the crack tip. The stress intensity factor amplitude is obtained using the same engineering algorithm (according to ASTM E647 standard) as the experimental data processing to obtain the Paris variation curve, as shown in Fig. 4f,g.

From the final results, the error in crack length is 3.95% for the MP plate for both experimental and numerical simulations. For the S + F shot-blasted plate, the error in crack length was 10.76%. It can be seen that the experimental and numerical simulation errors are minor and within acceptable limits, which means the numerical simulation is basically valid.

### Analysis of crack growth results

To investigate the influence of residual stress field on crack growth after the S + F process, the finite element model of the MP plate and S + F plates was established, and the finite element analysis results are shown in Fig. 3b,c. It was found that compared to the MP, the crack tip stress on the surface layer of the specimen decreased by about 40% after the S + F process. After the S + F process, small defects appeared on the surface of the specimens, which could easily provide a budding point for cracks. However, the residual stress depth and residual stress value of the specimens after shot peening process showed a significant increase compared to the untreated specimens. Based on Elber’s plasticity closure theory, the effective stress intensity factor decreases as the crack passes through the cratered region, making it easier for crack closure to occur compared to the untreated region. Under an applied alternating load, the crack will growth only when the effective stress intensity factor caused by the crack tip reaches the critical stress intensity factor of the material itself, which means the deeper residual stress field introduced by the forming process can slow down the fatigue crack growth.

To further obtain the effect law of the S + F process on crack growth, the experimental and numerical simulation results of Fig. 4d,e were summarized. The results are shown in Table 2. In comparison, after the S + F process, the final crack growth length and fatigue life of the specimen is consistent with the MP plate. By investigating the effects of large-size shot peening forming process on the test fatigue life as well as the residual stresses, Wang^24^ found that the shot peening forming process alone would substantially reduce the fatigue life of the specimens. The shot peening strengthening before shot peen forming process used in this paper can keep the fatigue life of the specimen in line with that of the machine-processing plate, which is a great improvement compared with the fatigue life of the specimen formed by shot peening alone. In terms of residual stress, Wang^27^ found that the size and depth of the residual stress in the specimen increased with the increase of the shot peening size, which is the same as the test law obtained from the tests in this paper. From the comparison of Paris curves of MP plate and S + F plate in Fig. 4f,g, it can be seen that in the early stage of crack growth, the crack growth rate of S + F plate does appear to be elevated compared to that of MP plate, and shows large fluctuations. After observing the stress intensity factor amplitude corresponding to the location of the crack fluctuation initiation point and back-propagating the crack location at that point, it was found that the fluctuations all occurred near the crack growth to the forming crater. The crack growth rate decreases temporarily near the forming crater and increases rapidly during the passage through the forming crater, leading to fluctuations in the previous crack growth rate. However, with the gradual weakening of the overall stress intensity factor gain amplitude, the crack growth rate of the late S + F plate remains at the same level as that of the MP plate.

**Table 2:** Results of experiments and numerical simulations.

Test project	Method	a/mm	N/r
MP S + F	Experiment	20.83 22.33	143,016 141,021
MP S + F	Numerical simulation	20.01 19.92	124,013 126,859

## Discussion

### “Fluctuation phenomenon” after S + F

As shown in Fig. 4f,g, compared with the MP plate, the crack growth rate of the S + F plate increases in the early stage of the strengthening crater, and decreases in the forming crater, making the “fluctuation phenomenon” of the shot peening curve occur.

**Figure 5:**
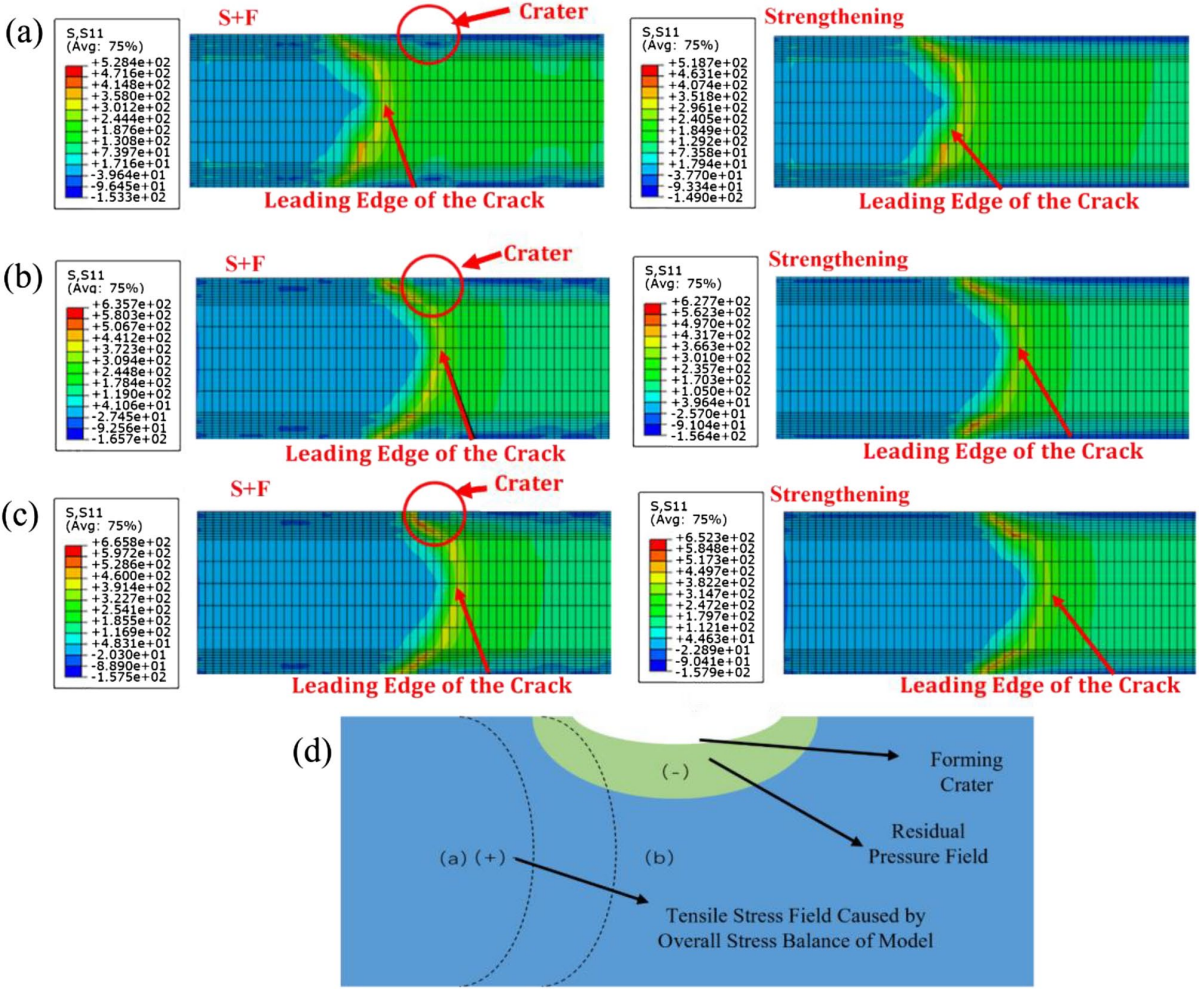
Tip stresses in different relative positions: (**a**) Before residual stress-affected area; (**b**) Enters residual stress-affected area; (**c**) Enters crater geometric boundary area; (**d**) Mechanism of crack growth effects.

In order to investigate the “fluctuation phenomenon” that happens after the crack passing through the forming crater, we only introduce the shot peening strengthening residual stress field into the model and then compare it with the model using the S + F process. The results of comparative analysis of the crack tip stress field at different stages of crack growth are shown in Fig. 5a–c. Through the comparison of the relative position of the crack leading edge and the formed crater, it can be found that when the leading edge of the crack is outside the influence range of the residual stress field of the crater, the crack tip stress level under the two processes is basically the same. The occurrence of fluctuation locations were observed near the forming crater in both the experimental and numerical simulations of the Paris result curves, so the analysis of the “fluctuation phenomenon” was studied with the forming process as a variable. The changes in the crack tip stress field of the single shot peening strengthening process and S + F process crack growth to different stages were also analyzed. According to Fig. 5d, the mechanism of the effect of forming crater on the crack growth rate can be concluded that when the crack expands near the forming crater, it will enter the tensile stress field and residual compressive stress field successively, which will cause the crack growth rate to change. However, after the crack continues to expand forward until it leaves the influence of the forming crater, both models are only affected by shot peening strengthening, leading to the basically same crack tip stress level. When the crack length reaches 8.13 mm, the crack tip enters the residual stress influence area of the forming crater. Compared with the model that only introduces the residual stress field of the strengthening process, the stress level of the crack tip after the S + F process is reduced by about 18.7%. Moreover, when the crack continues to expand forward to 9.59 mm, the stress concentration occurs when the leading edge of the crack touches the physical geometric boundary of the crater. Under the combined effect of the formation residual stress field, the crack stress field of the S + F process is increased by about 2.1% compared with the strengthening process, eventually leading to an increase crack growth rate when passing through the crater.

The mechanism of the influence of the formed crater on crack growth is shown in Fig. 5d. When the leading edge of the crack is located at the (a) position, the crack growth is affected by the tensile stress field caused by the overall stress balance of the model, making the overall stress intensity factor amplitude and the crack growth rate increase. When the crack is extended to position (b), after the crack is extended to the area of the formed crater, the crack expansion is inhibited due to the influence of the residual pressure stress of the secondary impact, which eventually gives rise to the “fluctuation phenomenon” of early crack extension near the crater after using the S + F process.

### *“Intersection phenomenon” after S* + *F*

From Fig. 4c, it is found that before a = 12.41 mm, the crack growth rate of the MP plate is lower than that of the S + F plate; after a = 12.41 mm, the crack growth rate of the MP plate is greater than that of the S + F plate, so the “intersection phenomenon” in the fitting curve occurs. Then, the overall stress intensity factor is introduced and a finite element model is developed to investigate “the intersection phenomenon” in the curve.

In the current research on shot peening, it is generally believed that the residual compressive stress in the surface layer can counteract the external loading effect, while the grain refinement can improve the strength of the material and the resistance to dislocation slip deformation. The results of the experiments and numerical simulations in this paper show that the introduction of the S + F process not only leads to fluctuations in the crack growth rate, but also to an increase in the crack growth rate of the pre-phase. The crack growth rate is controlled by the overall stress intensity factor of the crack tip, which can be divided into the external load stress intensity factor (${K}_{load}$) and residual stress field stress intensity factor (${K}_{res}$)^41^. Based on the VCCT, the crack tip energy release rate can be directly obtained from the crack tip support reaction force and the tension displacement. Moreover, with the linear elastic fracture mechanics, the relationship between the stress intensity factor K and the crack tip energy release rate can directly calculate the overall stress intensity factor of the crack tip.

Compared to the MP plate, due to the increase in residual tensile stress in the area caused by the overall stress balance of the S + F specimen, the overall stress intensity factor in the area, which is not affected by shot peening forming, is significantly higher in the specimen after the S + F process. Additionally, the overall stress intensity factor at the crack tip decreases with increasing crack length inside the formed crater region due to the deeper and larger residual compressive stress field introduced by the secondary impact of shot peening forming.

**Figure 6:**
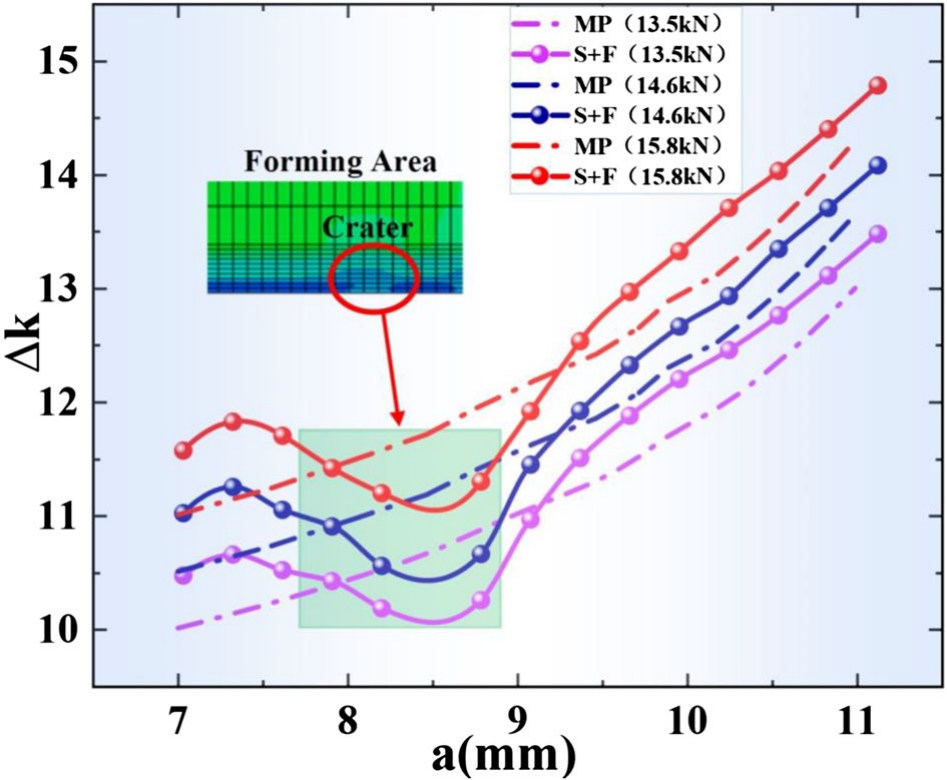
Overall stress intensity factor at different crack lengths.

In order to further investigate the “intersection phenomenon” of crack growth rate and to study the influence law of load level on the overall stress intensity factor, finite element models with external loads of 13.5 kN, 14.6 kN and 15.8 kN were established. The final results are shown in Fig. 6.

It can be seen from Fig. 6 that for the same crack length, the overall stress intensity factor of the crack tip increases with the external load level. After the S + F treatment, the overall stress intensity factor appears to be increased in the early stage of crack growth compared to the MP plate. However, the magnitude of the gain gradually decreases with increasing crack length, and the overall stress intensity factor of the S + F plate finally remains at the same level as the MP plate.

This phenomenon also shows that after shot peening strengthening, due to the combined effect of the residual compressive stress field caused by the strengthening process and the tensile stress field generated by the overall stress balance of the forming process, the crack growth in the early unformed crater area shows an increase in the overall stress intensity factor compared to the MP plate, which in turn delivers a faster crack growth rate. In contrast, after entering the crater area, the overall stress intensity factor decreases to a level below the MP plate due to the residual compressive stress of the forming process.

The stress intensity factor in the horizontal coordinate of the crack growth rate test is based on the engineering algorithm. The crack tip stress intensity factor is related to specimen's geometric shape, geometry, and crack length. However, in the same test, it is only associated with the crack length, so the Paris curve in the engineering algorithm describes the relationship between the crack length and the overall stress intensity factor. The results of the above numerical analysis explain the “intersection phenomenon” of the fitted Paris curves obtained in the crack growth rate test. The overall stress intensity factor such as the crack growth rate is no longer a linearly correlated amount of crack length after the S + F process, and the overall stress intensity factor appears to be elevated compared to the MP plate when the cracks are short in the early stage and converges with the MP plate in the later stage. When a linear fit is used, the intersection with the MP plate Paris curve will appear. The S + F process does not change the Paris curve of the material, but indirectly changes the Paris fitting curve under the engineering algorithm by changing the overall stress intensity factor.

## Conclusions

In this work, we investigated the effect of shot peening strengthening before shot peen forming process on the crack growth rate of 2024 aluminum alloy and the mechanism of the effect of large shot peening forming crater on crack growth by shot peening through experiments and numerical simulations. The following conclusions are drawn below.

1. After shot peening strengthening before shot peen forming process, the stress at the crack tip of the specimen surface was reduced by 40%, which effectively suppressed the crack growth of the specimen surface due to the impact of the projectile. At the same time, the crack growth rate of the shot peening plate remained the same as that of the machine-processing plate after a short fluctuation.
2. After the shot peening strengthening before shot peen forming process, the overall stress balance of the plate would lead to an approximate 4.5% increase in the overall stress intensity factor of the non-crater area, which caused higher crack growth rate of the shot peening strengthening before shot peen forming plate than that of the machine-processing plate in the non-crater area. When the crack entered the forming crater area, the overall stress intensity factor decreased by about 9.8% by the residual compressive stress. The crack growth rate of the shot peening plate dropped to a lower level than that of the machine-processing plate, which is why the rate of the crater area fluctuated and the “fluctuation phenomenon” gradually disappeared as the amplitude of the stress intensity factor at the tip of the crack increases.
3. The shot peening strengthening before shot peen forming process results in a formed crater coverage of about 30%, and the crack is mainly expanded by the non-crater area, so the early crack growth rate is faster. However, the gain amplitude of the residual stress gradually decreases with the increase of the stress intensity factor amplitude, and the crack growth rate remains almost the same as that of the machine-processing plate. This is the reason for the special intersection phenomenon appears in the Paris fitting curve.

In this paper, by deeply studying and exploring the effect of shot peening strengthening before shot peen forming process on the crack growth rate of 2024-T351 aluminum alloy, we sincerely wish to provide help and guidance for the development of effective cracking process of 2024-T351 aluminum alloy.

## Supplementary Information

Supplementary Information.

## Data Availability

The datasets generated during and/or analysed during the current study are available from the corresponding author on reasonable request, and the relevant data is uploaded to the additional file.
