# Supplementary material for: Study on the influence of shot peening strengthening before shot peen forming on 2024-T351 aluminum alloy fatigue crack growth rate
Source: Sci Rep. 2023 Mar 31;13:5313. doi: 10.1038/s41598-023-32616-2 (PMC10066396; doi:10.1038/s41598-023-32616-2)
Supplement: Supplementary file 1 — Supplementary Information. [file 41598_2023_32616_MOESM1_ESM.docx]

**Supplementary Information**

**Study on the influence of shot peening strengthening before shot peen forming on 2024-T351 aluminum alloy fatigue crack growth rate**

Guowei Li^a^, Zhicheng Dong^a^, Tianhao Luo^b,d,^, Heyuan Huang^b, c,^ *

*^a^ School of civil aviation, Northwestern Polytechnical University, Xi’an, China;*

*^b^ School of Aeronautics, Northwestern Polytechnical University, Xi’an, China;*

*^c^Research & Development Institute of Northwestern Polytechnical University in*

*Shenzhen, Shenzhen, Guangdong, China;*

*^d^Institute of Optics and Electronics, Chinese Academy of Sciences, Chengdu, China*

***Corresponding author**: Heyuan Huang

Correspondence details: No. 127, Youyi West Road, Beilin District, Xi'an, Shaanxi, China.

E-mail address: [huangheyuan@nwpu.edu.cn](mailto:huangheyuan@nwpu.edu.cn)

Model meshing diagram:


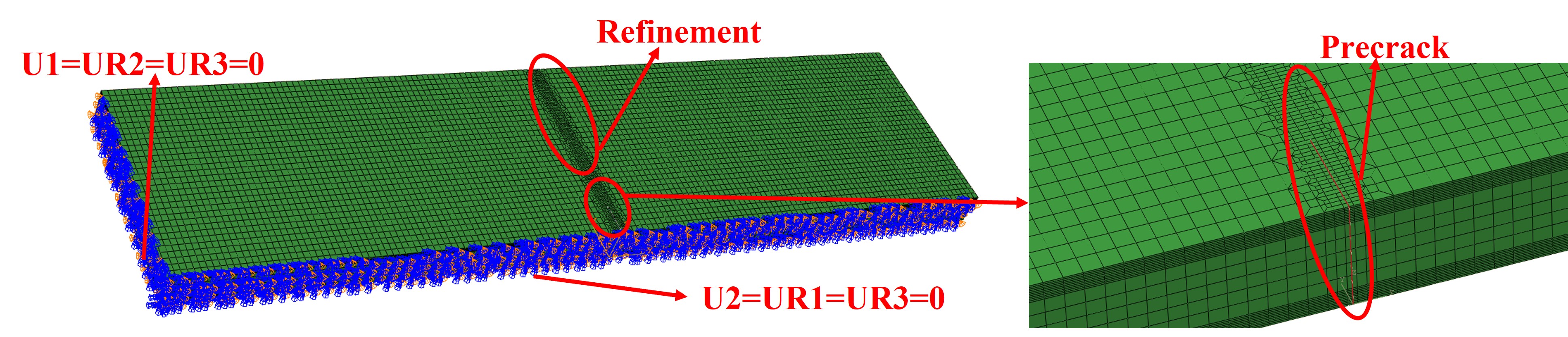


Figure S1. Model meshing diagram.

Plugins and FINN results:


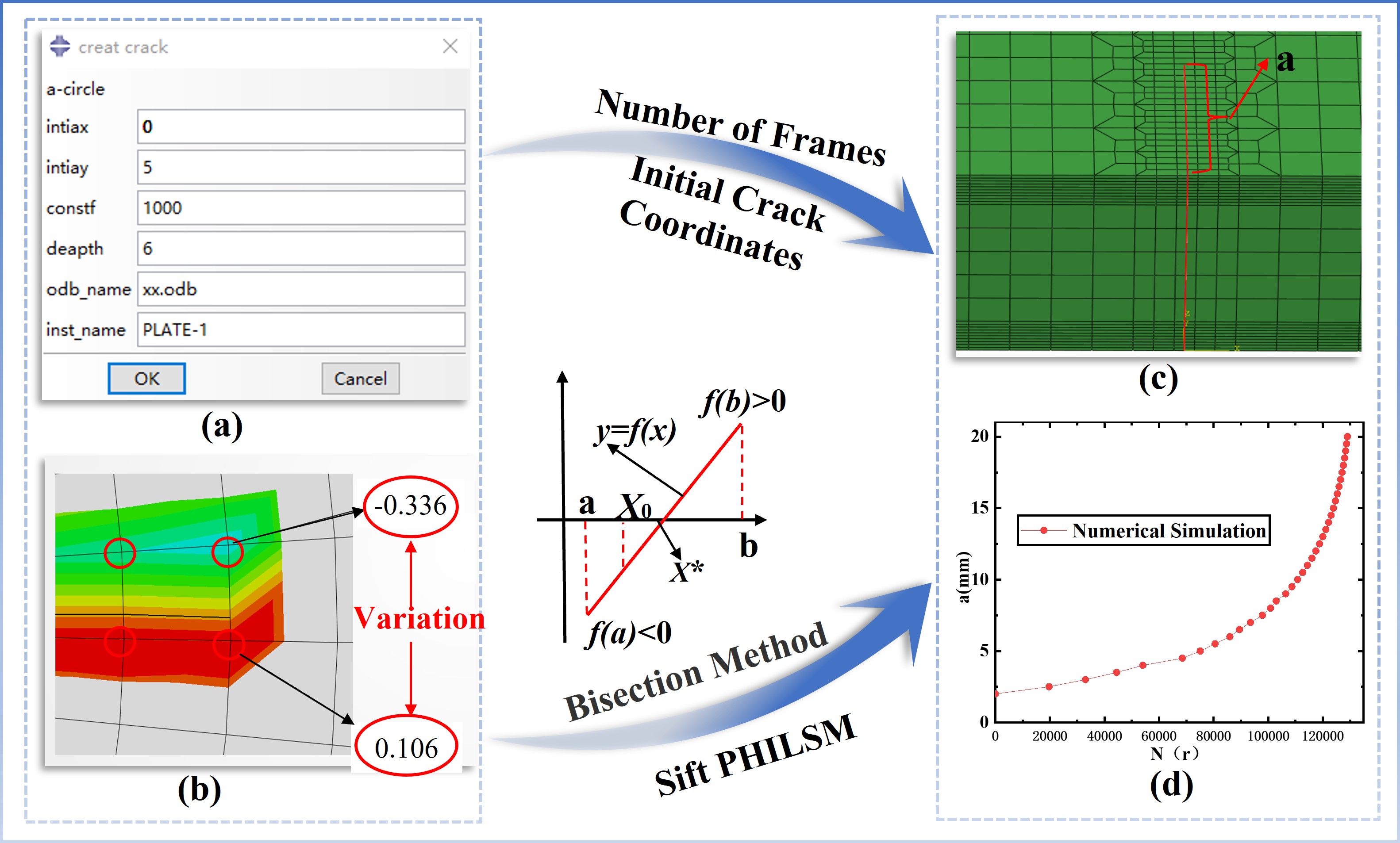


Figure S2. Plugins and FINN results:(a) Plugin interface;(b) Schematic diagram of PHILSM variations;(c) Crack length;(d) Finite element calculation result.

Test data


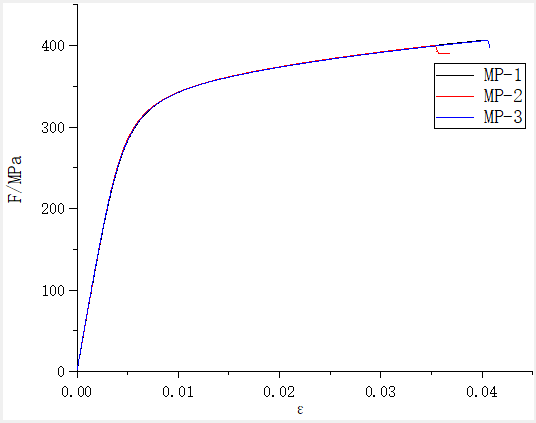


FigureS3 Tensile test stress-strain curve


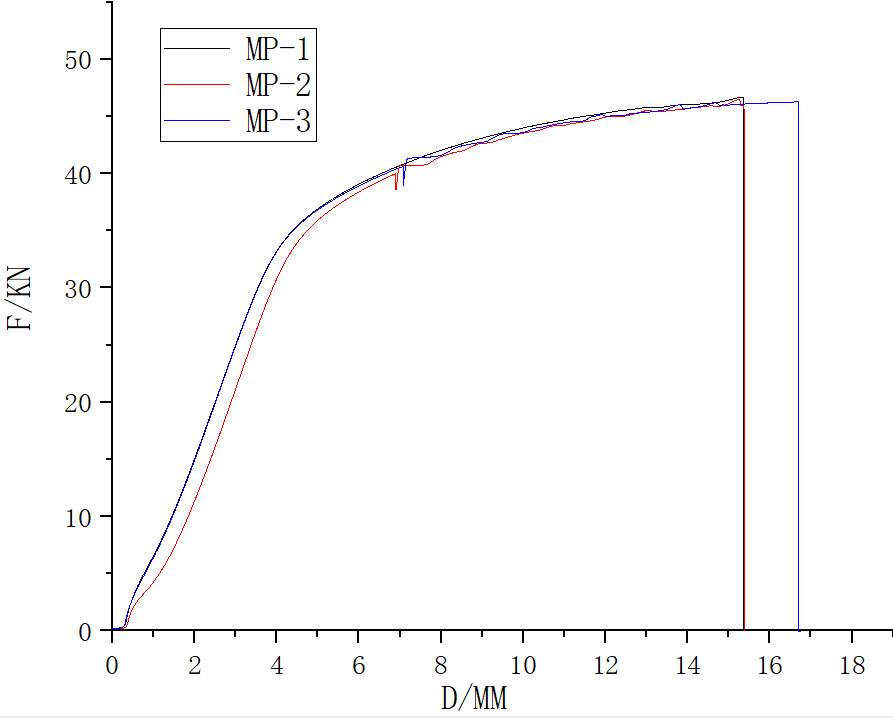


FigureS4 Tensile test load-displacement curve

TableS1 Tensile test result

| Test piece type | Elongation after break /% | Cross-sectional shrinkage /% |
| --- | --- | --- |
| MP | 14.66 | 12.63 |
| S+F | 8.21 | 8.52 |


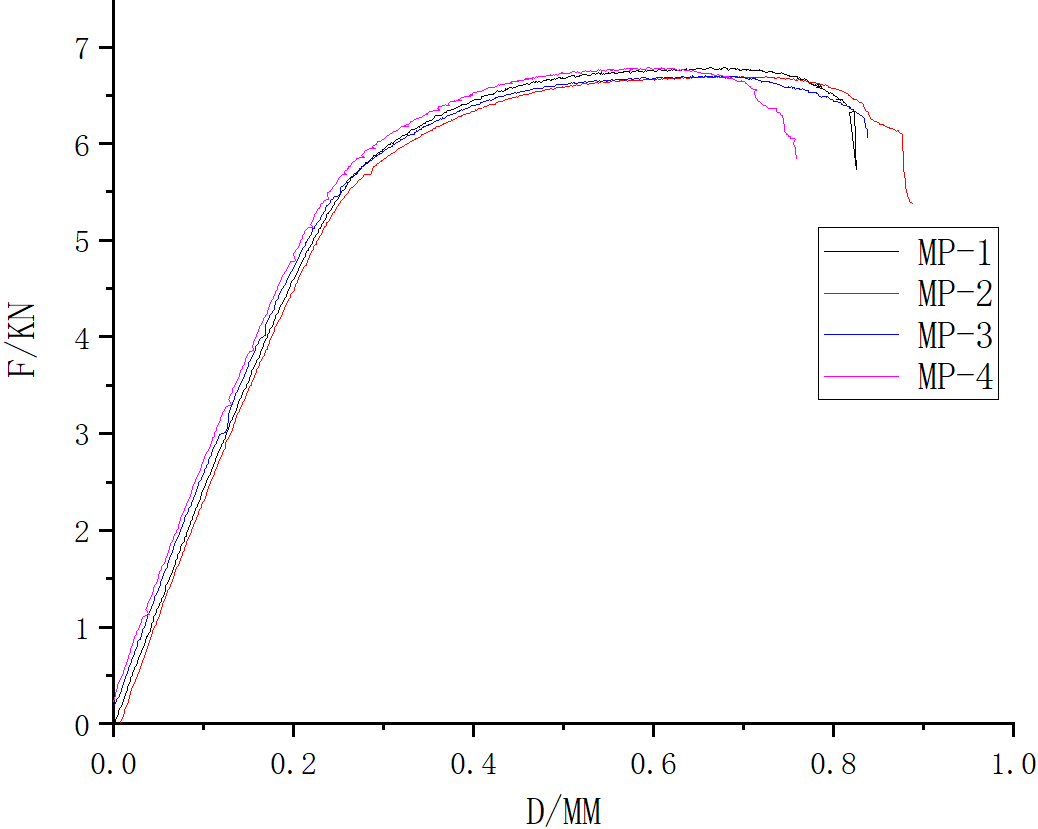


FigureS5 Shear test load-displacement curve


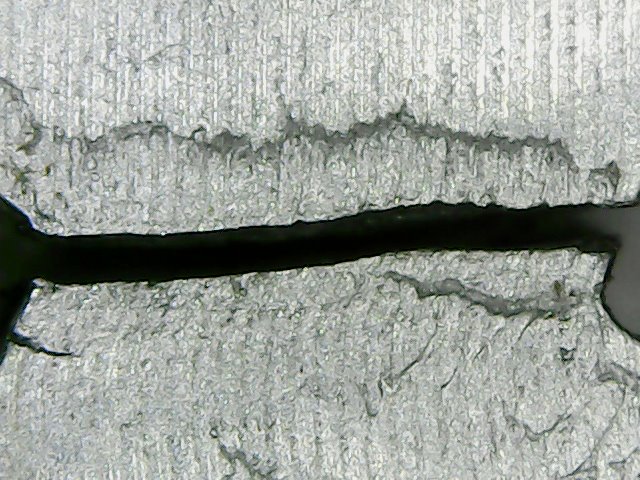

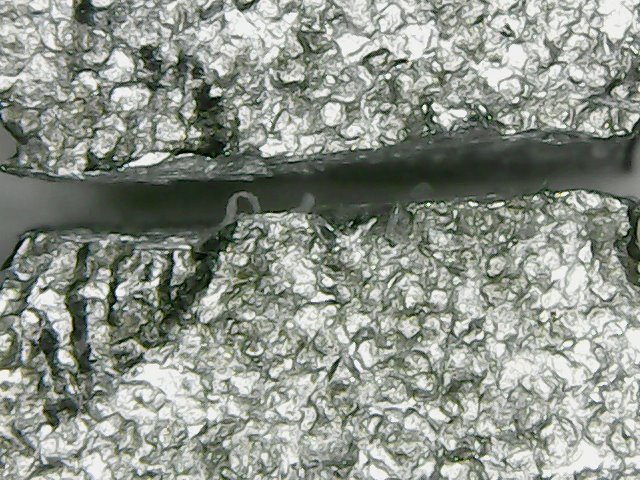


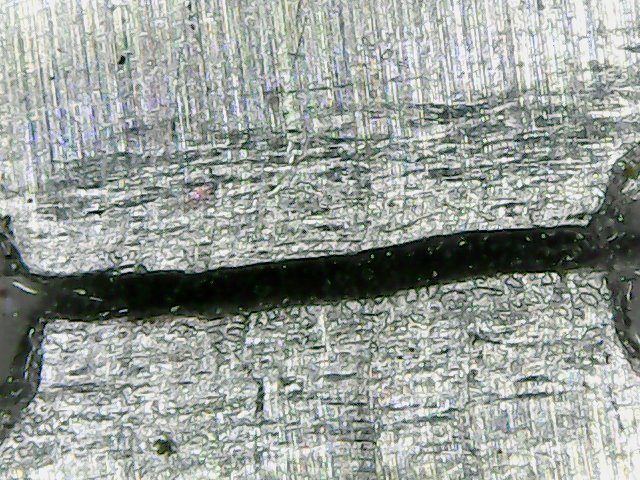

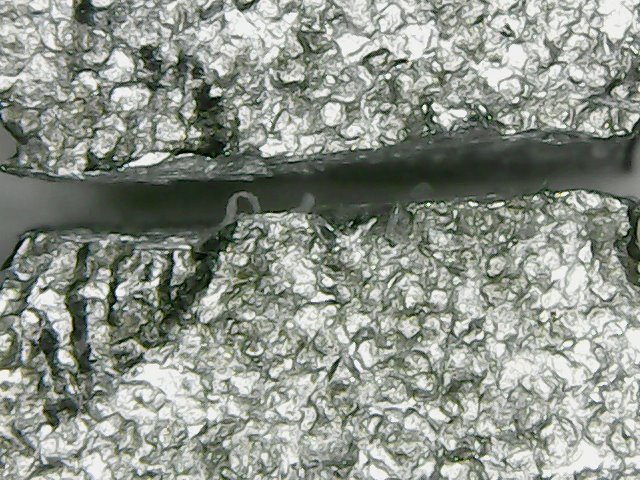
FigureS6 Shear fracture shape

TableS2 Residual Stress

| Distance | Shot peening  Strengthening | Shot peening  forming |
| --- | --- | --- |
| 0 | -150.29 | -230.94 |
| 50 | -210.74 | -245.45 |
| 100 | -216.43 | -252.08 |
| 150 | -198.09 | -225.34 |
| 200 | -171.35 | -199.02 |
| 300 | -124.92 | -154.03 |
| 400 | -96.82 | -136.21 |
| 600 | -46.14 | -99.11 |
| 800 | -15.37 | -50.38 |
| 1000 | / | -20.25 |

TableS3 Test piece size measurement results

| 编号 | W/mm | B/mm | | | B-Average value/mm |
| --- | --- | --- | --- | --- | --- |
| MP-1 | 75.03 | 6.06 | 6.04 | 6.07 | 6.06 |
| MP-2 | 75.01 | 6.04 | 6.06 | 6.04 | 6.05 |
| MP-3 | 75.05 | 6.01 | 6.02 | 6.02 | 6.02 |
| MP-4 | 75.05 | 6.03 | 6.05 | 6.06 | 6.04 |
| MP-5 | 75.04 | 6.05 | 6.04 | 6.05 | 6.04 |
| MP-6 | 75.05 | 6.01 | 6.01 | 6.02 | 6.01 |
| S+F-1 | 75.34 | 6.06 | 6.09 | 6.10 | 6.08 |
| S+F -2 | 75.33 | 6.09 | 6.07 | 6.04 | 6.07 |
| S+F -3 | 75.37 | 6.05 | 6.05 | 6.06 | 6.05 |
| S+F -4 | 75.35 | 6.09 | 6.13 | 6.07 | 6.10 |
| S+F -5 | 75.28 | 6.04 | 6.05 | 6.03 | 6.04 |
| S+F -6 | 75.15 | 4.04 | 4.11 | 4.03 | 4.06 |

TableS4 Fatigue crack growth data of S+F

| n | a-A/mm | a-B/mm | a-C/mm | a-D/mm | a Average /mm |
| --- | --- | --- | --- | --- | --- |
| 0 | 0.6 | 0.6 | 2.2 | 2.1 | 1.375 |
| 8850 | 1 | 1.3 | 3 | 2.5 | 1.95 |
| 11083 | 1.1 | 1.6 | 3.4 | 2.5 | 2.15 |
| 15065 | 1.2 | 1.8 | 3.7 | 2.7 | 2.35 |
| 19645 | 1.4 | 2.3 | 4.2 | 2.8 | 2.675 |
| 23818 | 2.2 | 2.7 | 4.7 | 2.8 | 3.1 |
| 27053 | 2.4 | 2.9 | 5 | 2.9 | 3.3 |
| 30027 | 2.9 | 3.2 | 5.2 | 3 | 3.575 |
| 33164 | 3 | 3.5 | 5.3 | 3.1 | 3.725 |
| 36101 | 3.1 | 3.8 | 5.5 | 3.2 | 3.9 |
| 39196 | 3.2 | 4 | 5.6 | 3.2 | 4 |
| 42102 | 3.3 | 4.3 | 5.6 | 3.3 | 4.125 |
| 45132 | 3.6 | 4.5 | 5.8 | 3.4 | 4.325 |
| 48067 | 3.7 | 4.7 | 6.1 | 4.6 | 4.775 |
| 51107 | 3.8 | 4.7 | 6.7 | 5.4 | 5.15 |
| 53323 | 3.9 | 5 | 7 | 5.7 | 5.4 |
| 55638 | 4.3 | 5 | 7.4 | 6 | 5.675 |
| 58105 | 4.5 | 5 | 7.8 | 6.5 | 5.95 |
| 60147 | 4.8 | 5 | 8.1 | 6.8 | 6.175 |
| 62108 | 5 | 5.1 | 8.3 | 7.2 | 6.4 |
| 64021 | 5 | 5.1 | 8.6 | 7.4 | 6.525 |
| 66554 | 5.1 | 5.1 | 9 | 7.8 | 6.75 |
| 69377 | 5.1 | 5.3 | 9.6 | 8.2 | 7.05 |
| 71471 | 5.3 | 5.5 | 10 | 8.5 | 7.325 |
| 73478 | 5.3 | 5.8 | 10.2 | 8.7 | 7.5 |
| 75738 | 5.5 | 7 | 10.7 | 8.9 | 8.025 |
| 77625 | 7.4 | 8.4 | 11.2 | 9 | 9 |
| 79126 | 7.9 | 9.2 | 11.3 | 9 | 9.35 |
| 80359 | 8.2 | 9.5 | 11.6 | 9 | 9.575 |
| 81571 | 8.4 | 10 | 11.9 | 9 | 9.825 |
| 82764 | 8.6 | 10.2 | 12 | 9 | 9.95 |
| 84178 | 8.7 | 10.5 | 12.1 | 9 | 10.075 |
| 87595 | 8.7 | 10.5 | 12.4 | 9.3 | 10.225 |
| 90159 | 8.7 | 10.5 | 13 | 9.3 | 10.375 |
| 93096 | 8.7 | 10.7 | 13.7 | 10 | 10.775 |
| 95119 | 9 | 11 | 14.3 | 10.7 | 11.25 |
| 96572 | 9.3 | 11.3 | 14.5 | 11.4 | 11.625 |
| 98226 | 9.5 | 11.3 | 15 | 11.8 | 11.9 |
| 99895 | 10 | 12.5 | 15.2 | 12.2 | 12.475 |
| 101073 | 11 | 13 | 15.5 | 12.8 | 13.075 |
| 102077 | 11.5 | 13.5 | 15.7 | 13 | 13.425 |
| 103173 | 12 | 13.8 | 15.8 | 13.5 | 13.775 |
| 105092 | 12.8 | 14.5 | 16.3 | 14 | 14.4 |
| 106086 | 13.4 | 15 | 16.5 | 14.5 | 14.85 |
| 107091 | 13.8 | 15.4 | 16.8 | 15.1 | 15.275 |
| 108104 | 14.2 | 15.9 | 17.2 | 15.6 | 15.725 |
| 109631 | 14.9 | 16.5 | 17.9 | 16.2 | 16.375 |
| 110680 | 15.1 | 17.3 | 18.5 | 17 | 16.975 |
| 111821 | 15.3 | 18 | 19 | 17.4 | 17.425 |
| 112903 | 16 | 18.7 | 19.8 | 18.1 | 18.15 |
| 113885 | 17 | 19.3 | 20.7 | 18.8 | 18.95 |
| 114572 | 17.4 | 19.7 | 21.2 | 19.2 | 19.375 |
| 115314 | 17.9 | 20.4 | 22 | 19.4 | 19.925 |
| 116052 | 18.2 | 20.7 | 22.6 | 19.6 | 20.275 |
| 116867 | 18.4 | 21 | 23.6 | 19.8 | 20.7 |
| 117721 | 19.2 | 22 | 24.5 | 20.4 | 21.525 |
| 118443 | 20 | 22.4 | 25.5 | 21.3 | 22.3 |
| 119208 | 21.2 | 23.3 | 26.5 | 22.4 | 23.35 |
| 120039 | 22.5 | 24.3 | 27 | 23.3 | 24.275 |
| 120748 | 23 | 25.5 | 27.8 | 24.4 | 25.175 |
| 121281 | / | / | / | / | / |

TableS5 Fatigue crack growth data of MP

| n/ | a-A/mm | a-B/mm | a-C/mm | a-D/mm | a Average /mm |
| --- | --- | --- | --- | --- | --- |
| 0 | 0.4 | 0.2 | 2 | 1.8 | 1.1 |
| 6015 | 0.5 | 0.4 | 2.2 | 2.1 | 1.3 |
| 12017 | 0.7 | 0.5 | 2.5 | 2.4 | 1.525 |
| 17012 | 1.3 | 0.7 | 2.9 | 2.6 | 1.875 |
| 21047 | 1.5 | 0.8 | 3.1 | 2.8 | 2.05 |
| 25051 | 1.7 | 0.9 | 3.4 | 2.9 | 2.225 |
| 29077 | 1.7 | 1 | 3.7 | 2.9 | 2.325 |
| 33031 | 2.1 | 1.1 | 4.1 | 3 | 2.575 |
| 36023 | 2.3 | 1.1 | 4.1 | 3 | 2.625 |
| 40032 | 2.4 | 1.1 | 4.3 | 3 | 2.7 |
| 44062 | 2.6 | 1.2 | 4.5 | 3.2 | 2.875 |
| 48057 | 2.7 | 1.4 | 4.6 | 3.5 | 3.05 |
| 52008 | 2.9 | 1.6 | 4.6 | 3.6 | 3.175 |
| 56059 | 3 | 1.7 | 4.8 | 3.8 | 3.325 |
| 59058 | 3 | 1.8 | 4.9 | 4 | 3.425 |
| 62026 | 3.2 | 1.9 | 5.2 | 4.5 | 3.7 |
| 65014 | 3.4 | 2 | 5.4 | 4.6 | 3.85 |
| 68039 | 3.4 | 2 | 5.5 | 5.2 | 4.025 |
| 71039 | 3.6 | 2.1 | 5.8 | 5.6 | 4.275 |
| 74021 | 3.7 | 2.4 | 5.9 | 5.8 | 4.45 |
| 77070 | 4 | 3 | 6.5 | 6 | 4.875 |
| 79518 | 4.9 | 3.5 | 6.8 | 6.3 | 5.375 |
| 82048 | 5.5 | 3.7 | 7 | 6.3 | 5.625 |
| 85051 | 5.7 | 4.1 | 7.1 | 6.4 | 5.825 |
| 88036 | 6 | 4.3 | 7.4 | 6.7 | 6.1 |
| 91064 | 6.4 | 4.6 | 7.9 | 7 | 6.475 |
| 93577 | 6.5 | 4.9 | 8.1 | 7 | 6.625 |
| 96546 | 6.6 | 5 | 8.3 | 7.4 | 6.825 |
| 99537 | 6.7 | 5 | 8.3 | 7.5 | 6.875 |
| 102515 | 7 | 5.3 | 8.5 | 8 | 7.2 |
| 105037 | 7.2 | 5.6 | 8.7 | 8.5 | 7.5 |
| 107560 | 7.4 | 5.9 | 8.8 | 9 | 7.775 |
| 110049 | 7.7 | 6.2 | 9.2 | 9.4 | 8.125 |
| 112544 | 8.1 | 6.4 | 9.5 | 10 | 8.5 |
| 115051 | 8.8 | 7.2 | 9.8 | 10.4 | 9.05 |
| 117539 | 9 | 7.5 | 10.4 | 10.6 | 9.375 |
| 120022 | 10 | 8.3 | 11.2 | 11.3 | 10.2 |
| 122033 | 10.5 | 8.6 | 11.7 | 11.7 | 10.625 |
| 124534 | 11 | 9.9 | 12.5 | 12.4 | 11.45 |
| 126518 | 11.5 | 11.1 | 13.1 | 12.9 | 12.15 |
| 128519 | 12 | 12 | 13.5 | 13.6 | 12.775 |
| 130524 | 12.6 | 12.7 | 13.7 | 14.1 | 13.275 |
| 133023 | 13.3 | 13.7 | 14.4 | 15.1 | 14.125 |
| 135016 | 14.1 | 14.4 | 15 | 15.8 | 14.825 |
| 137043 | 15 | 15.1 | 16 | 16.9 | 15.75 |
| 138028 | 15.3 | 15.5 | 16.5 | 17.4 | 16.175 |
| 139021 | 15.8 | 15.7 | 17.1 | 17.8 | 16.6 |
| 140021 | 16.3 | 16.6 | 17.6 | 18.4 | 17.225 |
| 141022 | 17 | 17.3 | 18.6 | 18.8 | 17.925 |
| 142011 | 17.7 | 17.8 | 18.8 | 19.5 | 18.45 |
| 142045 | 18.3 | 18.5 | 19.5 | 20.2 | 19.125 |
| 142089 | 19.1 | 19.3 | 20.1 | 21.1 | 19.9 |
| 143016 | 20.1 | 20.3 | 21.2 | 21.6 | 20.8 |
